# Thermoelectric properties of *in-situ* plasma spray synthesized sub-stoichiometry TiO_2−x_

**DOI:** 10.1038/srep36581

**Published:** 2016-11-04

**Authors:** Hwasoo Lee, Su Jung Han, Ramachandran Chidambaram Seshadri, Sanjay Sampath

**Affiliations:** 1 Center for Thermal Spray Research, Stony Brook University, Stony Brook, NY11794-2275, USA

## Abstract

The thermoelectric properties of sub-stoichiometric TiO_2−x_ deposits produced by cascaded-plasma spray process are investigated from room-temperature to 750 K. Sub-stoichiometric TiO_2−x_ deposits are formed through *in-situ* reaction of the TiO_1.9_ within the high temperature plasma flame and manipulated through introduction of varying amounts of hydrogen in the plasma. Although the TiO_2−x_ particles experience reduction within plasma, it can also re-oxidize through interaction with the surrounding ambient atmosphere, resulting in a complex interplay between process conditions and stoichiometry. The deposits predominantly contain rutile phase with presence of Magneli phases especially under significantly reducing plasma conditions. The resultant deposits show sensitivity to thermoelectric properties and under certain optimal conditions repeatedly show Seebeck coefficients reaching values of −230 μV K^−1^ at temperatures of 750 K while providing an electrical conductivity of 5.48 × 10^3^ S m^−1^, relatively low thermal conductivity in the range of 1.5 to 2 W m^−1^ K^−1^ resulting in power factor of 2.9 μW cm^−1^ K^−2^. The resultant maximum thermoelectric figure of merit value reached 0.132 under these optimal conditions. The results point to a potential pathway for a large-scale fabrication of low-cost oxide based thermoelectric with potential applicability at moderate to high temperatures.

Thermoelectric materials can generate electrical voltage directly from a temperature gradient and therefore, have attracted much attention as a means to harvest electrical energy from waste heat^1^. Efficiency is determined by a dimensionless figure of merit (*ZT*), where *ZT* = (*S*^2^*σκ*^−1^)*T*, where *S*, *σ*, *κ*, and *T* are the Seebeck Coefficient, electrical conductivity, thermal conductivity, and absolute temperature, respectively^2^. Unfortunately, the *ZT* of conventional systems cannot be substantially increased because the three parameters are constrained due to fundamental effects. A large Seebeck coefficient needs low carrier concentration, which invariably results in low electrical conductivity while low Seebeck coefficient materials offer a high electrical conductivity. To date, the leading materials are Bi_2_Te_3_-based alloys^3^, PbTe^4^, PbSe^5^, SiGe^6^, Mg_2_X (X = Si, Ge, Sn)^7^, skutterudite^8^, clathrate^9^, Zintl^10^ and half-Heusler alloys^11^. However, most of these rare earth alloy-based thermoelectric materials, such as Bi_2_Te_3_ and PbTe, suffer from thermal and chemical instabilities, high toxicity, relatively low availability and high cost. The use of rare or toxic elements in these compounds further limits their large-scale commercial application. An additional aspect that has limited the use of thermoelectric is the difficulty associated with their manufacture and ease of conversion from materials to engineering devices.

Transition metal oxide materials have received attention as possible thermoelectric materials as they offer reasonable electrical conductivity and Seebeck coefficient, while being cost-effective, environmentally friendly, and available over a range of compositions. Oxides of cobalt, copper, manganese, molybdenum, rhodium, titanium, tungsten, vanadium and zinc offer wide range of electronic properties ranging from insulating to semiconducting and conducting^12^. Among the many available oxide materials, Na_x_CoO_2_ and Ca_3_Co_4_O_9_ exhibit the best *p*-type thermoelectric performance^13^. In contrast, *n*-type oxides demonstrating high TE performance comparable to *p*-type oxides have not yet been developed. Potential candidates for *n*-type oxide thermoelectric materials include perovskite-type SrTiO_3_ and CaMnO_3_ where Nb-doped SrTiO_3_ has the highest ZT^14^ value so far, but it is still relatively low from point of view of applications. There is much effort in the literature to improve thermoelectric performance of *n*-type oxide materials by engineering their morphology, doping and stoichiometry^15^.

TiO_2_ naturally occurs in three polymorphic forms which are anatase, rutile and brookite. Anatase and rutile are both tetragonal while brookite exhibits orthorhombic structure^16^. The room temperature *S* of bulk TiO_2_ is reported to be approximately −440 μV K^−1^, with *σ* on the order of 10^3^ S m^−1^, while the *κ* is ~5.75 W m^−1^ K^−1 ^17. Owing to high *κ*, the *ZT* in pure TiO_2_ is low (<0.03). For titanium oxides, a relatively wide range of oxygen content is viable, ranging from TiO to TiO_2_^18^. Therefore, oxygen deficiencies and corresponding crystal lattice defects can be easily induced with concomitant implications on electrical properties. In particular, sub-stoichiometric TiO_2−x_ has been explored as a potential thermoelectric material^19^. Lu *et al*. have demonstrated that TiO_1.95_ prepared by oxidizing and reducing from the Ti metal reveals *S* of −800 μV K^−1^ and *σ* of 55 S m^−1^ at 460 K^20^. Tsuyomoto *et al*. have reported that TiO_1.94_ with an orthorhombic crystal structure prepared by reducing the anatase in H_2_, exhibits a peak *S* of −518 μV K^−1^ and *σ* of 1.9 × 10^3^ S m^−1^ at 343 K^21^. They showed that both *S* and *σ* increased with temperature resulting in power factors (*PF)* of ~510 μW m^−1^ K^−2^ at 343 K and ~35 μW m^−1^ K^−2^ at 460 K, respectively. Furthermore, a single crystal of rutile TiO_2_ reduced to TiO_2−x_ via annealing in H_2_ elevated temperature at 1053 K shows exceptionally high *S* of up to 60000 μV K^−1^ at cryogenic temperatures of 10 K^22^, and interpreted the results preferably due to the phonon drag effect of the holes. Evidently, the introduction of oxygen vacancies in the TiO_2_ crystal lead to increase in carrier concentration resulting in the enhancement of *σ*. However, an excessive oxidation of TiO_2_ at elevated temperature lowered *σ* and *S,* resulting in low *PF* and *ZT*. Thus, obtaining a fabrication process for TiO_2−x_ should be chosen to maximize the high *PF* while maintaining the low thermal conductivity.

Sharma *et al*.^23^ observed that plasma-sprayed TiO_2_ deposits behave as *n*-type semiconductor with reasonable conductivities (1.76 × 10^3^ S cm^−1^) up to about 823 K, after which rapid re-oxidation occurs with concomitant increases in resistance. This was attributed to oxygen loss occurring during the spray process and subsequent trapping of the vacancy state through rapid solidification. Subsequently, Colmenares-Angulo *et al*.^24^ examined process-induced stoichiometric changes in plasma sprayed TiO_2_ deposits allowing tunability in electrical conductivity. There have been significant efforts to use thermal spray processes to obtain high anatase content TiO_2_ for potential applications such as for large area photo catalysts for decomposition of hydrocarbons and nitrogen oxides^25^ where TiO_2_ is extensively used for variety of industrial applications particularly in the form of films and coatings including optical, electrical, photocatalytic and tribological applications^26^.

In this study, the thermoelectric properties of plasma sprayed TiO_2−x_ are examined through variations *in-situ* reduction within the process. Plasma spray offers a range of conditions through not only the melt state of the material is affected, but also its stoichiometry associated with complex reactions between the high temperatures and its surrounding environments comprising initially of an active (reducing in presence of H_2_ and increase Seebeck coefficient) plasma and subsequently oxygen at the location of deposition^27^,^28^. The deposit itself comprises of layered “splats”, some porosity and cracking which can offer potential benefits (reduced thermal conductivity and increased Seebeck coefficient) and deleterious (reduced electrical conductivity) in the formed deposit. Through a systematic series of processing experiments coupled with specimen fabrication, the thermoelectric properties of the specimens were evaluated and optimized with promising initial results. An exhaustive parametric study using both conventional and cascaded plasma torches considering two types of powders; a near stoichiometric (TiO_1.9_) and highly reduced (TiO_1.7_). For brevity only cascaded plasma spray results with the TiO_1.9_ powder is reported as only these conditions showed promising results. Of importance is the uniform thermal fields of cascaded plasmas that reduces variabilities in both particle temperature and plasma-particle interactions^29^. Plasma spray is a flexible and scalable manufacturing processing technique capable of operating in an ambient environment and allows assembly of mesoscale multilayers both as blanket deposit and patterned structures with metals (as potential conductors), ceramics (as dielectric interlayers) and functional oxides^30^,^31^,^32^. In addition, the process allows for conformal deposition directly onto components potentially enabling direct fabrication of useful thermoelectric devices for exploiting applications including cooling and refrigeration^33^, energy harvesting from heat^34^, solar thermoelectric generators and radioisotope thermoelectric generators^35^, and sensors^36^.

## Results and Discussion

As noted in the methods section (end of paper), a nominally stoichiometric TiO_2_ industrially produced feedstock powder (measured to be TiO_1.90_) was used to produce plasma spray deposits. This powder is a plasma spray grade with particle sizes ranging from 8.6 to 28.9 μm. As noted in the introduction, as part of initial screening study, over 18 different iterations of spray conditions were attempted considering both conventional and cascaded plasma spray torches as well as high velocity combustion thermal spray systems. The cascaded plasma spray torches with its uniform thermal field yielded more homogeneous deposits with significant results and as such only data from this study is reported in the paper. In this case, the H_2_ content of the plasma was systematically varied to control the oxygen loss and thus stoichiometry and phase of the deposit. Three significant process conditions (Deposit A, B, and C) comprising of differences in plasma spray state and H_2_ content of the plasma gas are discussed in detail to and were analyzed for their phase, stoichiometry, microstructure and thermoelectric properties. This integrated strategy not only allows highlighting the significant finding but also establish the process-property relationships and to identify the contributing aspects that enhance the thermoelectric properties.

### Phase study and microstructure of the deposits

**Figure 1:**
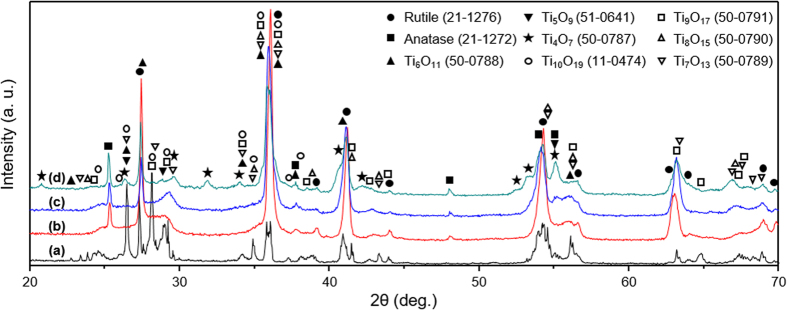
Powder XRD patterns of as-received powder and deposits. (**a**) Feedstock, (**b**) Deposit A, (**c**) Deposit B, and (**d**) Deposit C.

Figure 1 shows the XRD patterns of the starting feedstock powder and three deposits produced under different hydrogen content within the plasma indicating that diffraction peaks from the feedstock powder diminishes and broadens for as-sprayed deposits as they showed broad diffuse reflection in the 2θ positions near by the rutile peaks. The starting feedstock powder is primarily composed of rutile and Magneli phases (Ti_n_O_2n−1_ where n = 5, 6, 7, 8, 9, 10). The sprayed deposits retained the majority of the rutile phase along some of the Magneli phases (n = 5, 6) and trace amounts of anatase. Among the deposits, deposits A and B (with 0 and 3 L min^−1^ of H_2_) show Magneli phases with n = 5, 6 while deposit C (sprayed with 9 L min^−1^ of H_2_) shows Magneli phases with n = 4, 5 and 6. The increase in Magneli phases especially Ti_4_O_7_ is resultant from both the high enthalpy of the plasma at this condition and more reducing environment of the plasma.

As the H_2_ content in the plasma increases, not only the phase structure and crystallinity are affected, but there is deviation from stoichiometry due to the oxygen loss. Deposit A produced without any H_2_ in the plasma indicated a small gain in stoichiometry (TiO_1.93_) perhaps associated with interaction of the hot particle with oxygen in the environment at the deposition location^37^,^38^. Deposit C sprayed with 9 L min^−1^ of H_2_ showed significant loss of oxygen resulting in a stoichiometry of TiO_1.83_. Deposit B sprayed with 3 L min^−1^ of H_2_ in the plasma maintains the stoichiometry, but this again may be associated with competition of oxygen loss in the active portion of the plasma and regaining the oxygen at the deposition location due to comingling with atmospheric oxygen dampening the next changing in stoichiometry. Deposit B* sprayed with 6 L min^−1^ of H_2_ (included in Table 1) followed this trend with a stoichiometry of TiO_1.86_ confirming this operative mechanism.

**Figure 2:**
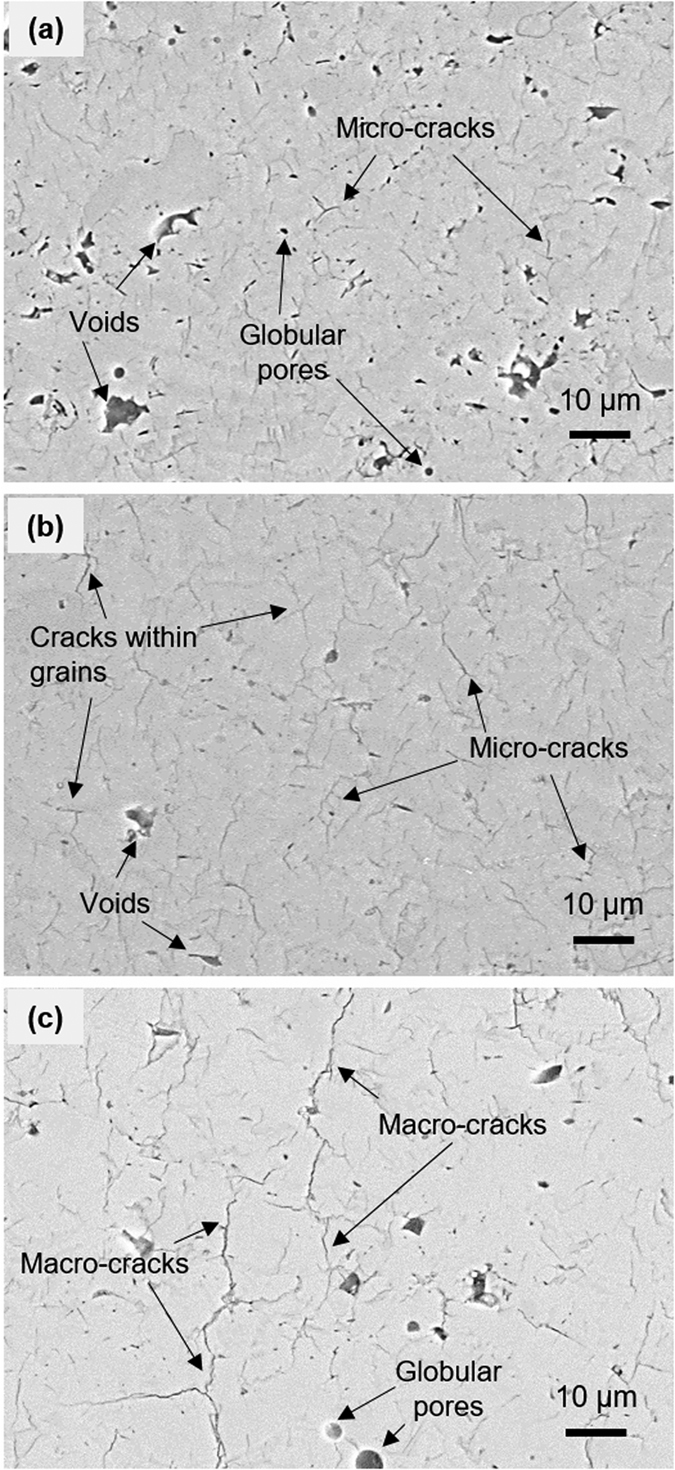
Cross-sectional SEM images of as-sprayed TiO_2−x_ deposits with 3.0 K magnification (**a**) Deposit A, (**b**) Deposit B, and (**c**) Deposit C.

Figure 2(a–c) presents the representative cross-sectional SEM micrographs of the as-sprayed deposits A, B and C processed under 0, 3, and 9 L min^−1^ of H_2_ conditions, respectively. The image of Fig. 2(a) shows deposit A with some porosity and micro-cracks within the lamellae. Since relatively lower temperatures are involved in the formation of deposit A, it consists of thinner splat layers with fewer and substantially narrower interlamellar pores. Figure 2(b) shows cross-section of denser deposit B with numerous micro-cracks. The image of Fig. 2(c) is similar to 2(b), but the deposit C now comprises of larger connected macro-cracks. The deposit C sprayed at higher plasma energy and particle temperature (Table 1) will lead to greater quenching stresses for the solidifying particle as well as higher substrate temperature (530 K for 9 L min^−1^ of H_2_ whereas 425 K for 0 L min^−1^ of H_2_) consequently leading to greater propensity of micro and macro-crack formation. Deposit B had relatively lower particle and substrate temperature resulting in reduced propensity for micro-cracking. This micro-cracking is attributed to the large quenching stresses associated with the rapid solidification of the depositing molten particles. The particles are exposed to the high thermal energy resulting in melting followed by rapid solidification which induces volume change and temperature change resulting in build-up of large tensile stress and which are relieved through cracking^39^,^40^. Deposit A at low thermal energy condition (0 L min^−1^ of H_2_) shows the lowest density (3.925 g cm^−1^) compared to those sprayed at higher temperature, and exhibits a somewhat porous microstructure. Deposit B and C show increase in density from 4.138 to 4.218 g cm^−1^, but also greater content of micro-cracks.

*In situ* beam curvature measurements were conducted to further quantify these microstructural observations via extracting the deposition stress and elastic modulus of the sprayed deposits following principles described in the literature^41^,^42^,^43^,^44^. This technique provides a volume averaged information about the microstructural integrity and interlamellar bonding and as such provides reliable information of regarding the nature of the deposits. Despite the higher enthalpy processing condition, deposit C showed lower modulus (139 GPa) and lower evolving stress (24 MPa) compared to the deposit A whose values were 160 GPa for modulus and 90 MPa for evolving stress, respectively. Typically, H_2_ imparts higher enthalpy to the plasma and thus higher particle temperature and this should generally produce higher density deposits. The lower evolving stress and lower modulus in deposition C confirm the microstructural observations in terms of observation of substantial micro- and macro-cracking at the higher H_2_ conditions. These cracks also lower the deposit integrity captured by the lower modulus of deposit C.

### Thermoelectric Properties

**Figure 3:**
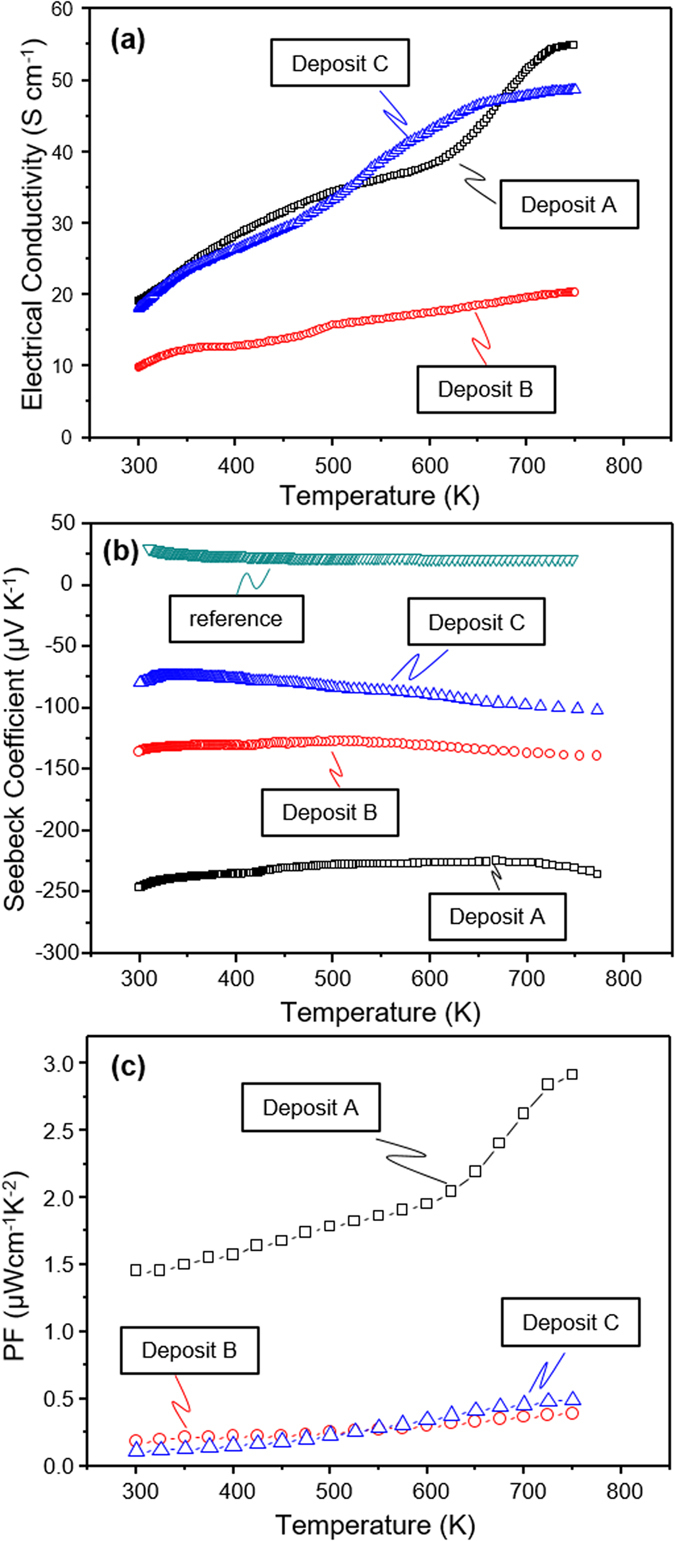
Temperature dependent electrical transport properties and thermoelectric power factors of TiO_2−x_ deposits, (**a**) electrical conductivity, *σ*, (**b**) Seebeck Coefficient, *S*, and (**c**) power factor, *PF*.

The electrical conductivity, *σ*, Seebeck coefficient, *S*, and power factor, *PF*, for the deposits are summarized in Fig. 3. The electrical conductivities for TiO_2−x_ deposits respond similar to a semiconductor following same temperature-dependent trend as reported in the literature^20^,^45^. However, for the deposit A sprayed without H_2_ in the plume with a resultant stoichiometry of TiO_1.93_ can observe an increase in slope at 620 to 730 K following which there is a small decrease. The first change in slope above 620 K is attributed to the thermal excitation of carriers, while the second is related to the phase transition from space group I4_1_/amd to the P4_2_/mnm^24^. The temperature dependence of the electrical conductivity for the deposit B was relatively weak and the value of the *σ* is significantly lower than that of the deposit A because of the micro-cracks within its microstructure. Deposit C shows electrical conductivity similar to that of deposit A despite presence of macro-cracks in the deposit, due to the significant presence of Ti_4_O_7_ phase. This confirms the greater conductivities of the deposits associated oxygen loss with larger deviation from microstructure and phase.

The Seebeck coefficients for the deposit A, B, and C were all negative, ranging from −73.5 μV K^−1^ to −246 μV K^−1^, and were measured −230, −139, −100 μV K^−1^ at 750 K, respectively, suggesting that the deposits are *n*-type conductors. Deposit A comprising of larger fraction of the rutile phase exhibited the largest Seebeck coefficient. As He *et al*.^17^ has reported, stoichiometry close to TiO_2_ resulted in an increase in the Seebeck coefficient where bulk rutile had Seebeck coefficient of −440 μV K^−1^. The power factor increased with increasing measurement temperature for all of the deposits and the highest power factor of 2.91 μW cm^−1^ K^−2^ at 750 K was achieved with deposit A.

The difference in the Seebeck coefficient is attributed to the presence of oxygen defects which is inversely proportional to the carrier density whereas electrical conductivity depends upon combination of oxygen vacancy, microstructure and crystallinity^23^. Increase in hydrogen content of the plasma can lead to greater oxygen loss and thus further phase reduction (Ti_4_O_7_) with associated increase in electrical conductivity and reduction in Seebeck coefficient as well. However, microstructural integrity can also contribute to the measure of electrical conductivity. Although the deposit A has a lower oxygen deficiency, the deposit does not have significant micro and macro-cracking. The deposit C has a relatively high conductivity due to presence of Ti_4_O_7_ phase^46^,^47^ despite the presence of macro-cracks. The deposit B has neither the extent of phase nor the microstructural benefit resulting in low electrical conductivity. The results point to the operative mechanisms and a processing approach to optimize the desired features.

### Thermal Conductivity

**Figure 4:**
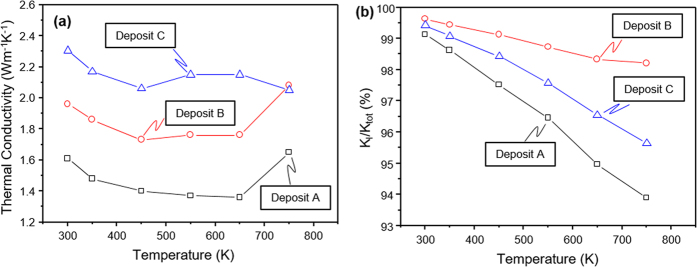
Temperature dependent thermal transport properties of TiO_2−x_ deposits, (**a**) thermal conductivity, *κ*, and (**b**) ratio of lattice thermal conductivity over total thermal conductivity, *κ*_*l*_/*κ*_*tot*_.

The total thermal conductivity for the TiO_2−x_ deposits ranged from 1.36 W m^−1^ K^−1^ to 2.17 W m^−1^ K^−1^ depending on the stoichiometry of the deposits and the temperature at which it was measured as shown in Fig. 4(a). The thermal conductivity of all three deposits decreased from room temperature to 450 K, but, surprisingly the behavior of the thermal conductivity changes above 450 K depending from the deposit because the difference in stoichiometry has effect on the phonon scattering^17^,^48^. A lowest total thermal conductivity value of 1.36 W m^−1^ K^−1^ at 650 K was measured from deposit A and the thermal conductivity value increased to 1.65 W m^−1^ K^−1^ at 750 K, which is relatively low compared to the literature^17^,^20^,^24^,^45^,^48^,^49^,^50^. Figure 4(b) shows the ratio of lattice thermal conductivity over total thermal conductivity of the TiO_2−x_ deposits. The *κ*_*l*_ was calculated by subtracting the *κ*_*e*_ from the total thermal conductivity. The electronic contribution was estimated by using the Wiedemann-Franz law^51^ and shows to be at most 0.1 W m^−1^ K^−1^ at 750 K in deposit A among the two other deposits and the ratio of *κ*_l_ to *κ*_tot_ are shown in Fig. 4(b). This ratio indicates that the total thermal conductivity is dominated by phonon transport. These results suggest that the extrinsic microstructural features such as density, micro- and macro-cracks, and porosity contributes higher than the intrinsic non-stoichiometry like defects and vacancies of the material^27^,^52^.

In this study we have only been able to report the thermal conductivity in the through-thickness direction as it is extremely difficult to reliably measure the conductivity in the in-plane direction for these thin deposits. It would be expected that the in-plane conductivity would be somewhat higher due to the lamellar nature of the sprayed material. However, the microstructure of sprayed layers is rather chaotic with successive assemblage of thousands of particles which eliminates some of the microstructural anisotropy. Chi *et al*.^52^ has reported this orientation dependence on thermal conductivity ranging from near equivalent up to 25% lower dependent on materials and process conditions. Recent studies conducted on very thick (25 mm) zirconia coatings allowing for measurements in both orientations confirm this earlier observation. Nevertheless, the process-property trends reported in this work should be consistent.

### Thermoelectric - Figure of Merit

**Figure 5:**
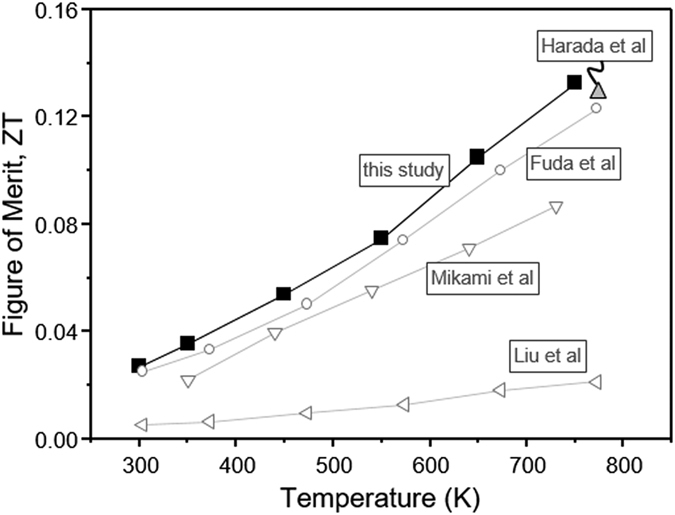
Temperature dependent dimensionless figure of merit, *ZT*, of deposit A and comparison of the references.

The calculated dimensionless figure of merit, *ZT* with reference from the literature is shown in Fig. 5. From this figure, it is apparent that the results reported here for deposit A are comparable or superior to the other reported data on the thermoelectric properties of TiO_2−x_^45^,^48^,^49^,^50^. It is clear that optimization of high Seebeck coefficient, high electrical conductivity and low thermal conductivity promotes enhanced thermoelectric property, in which the *ZT* value reached maximum at 0.132 at 750 K. Lastly, condition for deposit A was repeated in six separate processing trials to ensure repeatability with separate fabrication and measurements resulting in a highly repeatable *ZT* ranging from 0.123 to 0.133. In addition, complete multilayer devices incorporating multiple junction incorporating both series and parallel connections with Ni as a nominal *p*-type material showed scalable increase in voltage and current.

### Interplay among process-microstructure-thermoelectric properties

The results presented in this paper are significant from several points of view. First confirmation on the potential of sub-stoichiometric oxides such as TiO_2−x_ as alternative thermoelectric materials prepared through and industrial scale, layered manufacturing process. In Fig. 5, the results from this study are benchmarked with those reported in the literature indicating equivalent of better properties suggesting that plasma spray strategies may be of relevance to other oxide thermoelectric systems. Although plasma spray has been contemplated for thermoelectrics for many years, this is the first time that significant results have been achieved. This unusual result is related to non-obvious process-phase-microstructure-property relationships and as such warrants more detailed examination. In this section, we seek to distill these interrelations which will not only clarify the results presented in this paper but provide a framework for future developments for thermoelectric oxides in general and thermal sprayed functional oxides in particular.

**Figure 6:**
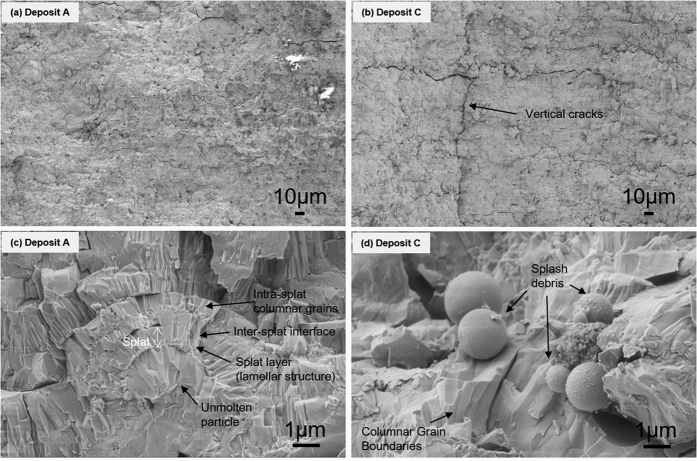
SEM images of fractured surface of TiO_2−x_ deposits: (**a**) Deposit A, (**b**) Deposit C with 1.0 K magnification; (**c**) Deposit A and (**d**) Deposit C with 30.0 K magnification. Deposit A shows unmolten particle while deposit C shows cracks and splash debris.

**Figure 7:**
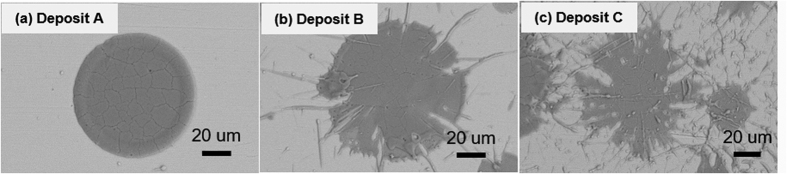
SEM images of TiO_2−x_ splats: (**a**) Deposit A, (**b**) Deposit B, and (**c**) Deposit C.

**Figure 8:**
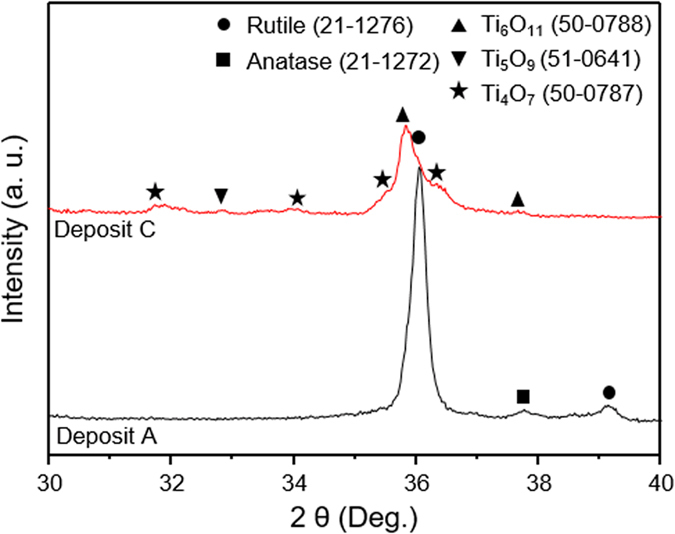
High resolution powder XRD patterns in the 30°–40° 2 θ range of the deposits A and C. Peaks are identified corresponding to the given JCPDS.

As noted, earlier although many iterations of process parameters and initial stoichiometry were examined as part of the larger study and among them, only condition for deposit A showed this relatively high thermoelectric figure of merit. By comparing condition for deposit A with B and C presented in this paper, key attributes that allow for high *ZT* can be identified:

- Consistent with contemporary knowledge, significant retention of the original rutile phase and reduced decomposition are beneficial to retain the high Seebeck coefficient in the TiO_2−x_ system. Both XRD and TGA results (Table 1) confirm that condition for deposit A retains higher fraction of the rutile phase and devoid of the decomposed Ti_4_O_7_ phase. This result is explained based both absence of H_2_ and lower average spray plume particle temperatures for deposit A. Lower average temperature indicates that some of the particles are likely unmelted and retained as such in the deposit. Detailed temperature distribution analysis from single particle measurements (not shown in the paper) confirms this to be case. The median temperature through this alternative measurement for deposit A is 2100 K compared to 2200 K for deposit B and 2340 K for deposit C. The absence of H_2_ in the plasma not only reduces the particle temperature but also suppresses the propensity for any inflight reduction, and potentially encourages interaction of the hot particle with the oxygen in ambient environment resulting in a small stoichiometry gain in the deposit A compared to deposit B and C.
- Under higher particle temperature and H_2_ rich conditions, loss of stoichiometry and presence of Ti_4_O_7_ Magneli phase are favored which suppress the Seebeck coefficient, but concomitantly offer higher electrical conductivity.
- Condition for deposit A is unusual in that it not only retains the Seebeck but also has high electrical conductivity with no significant deviation in thermal conductivity resulting in high *ZT*. To understand this unusual result, one needs to look at the microstructure more critically. As shown in Fig. 2, deposit A does not have significant micro or macro-cracks which can impede carrier flow. But beyond this obvious difference, more nuanced differences are seen through additional SEM analysis of fracture cross-sections and examining single isolated droplets on polished substrates. This is shown in Fig. 6 comparing low and high magnification images of deposit A and C. Low magnification images confirm the presence of both vertical and horizontal cracks in deposit C. The higher magnification (30,000 X) images provide additional insights. Deposit A shows relatively clean fracture surface with good microstructural integrity and reasonable connectivity among the splats. The image also shows presence of unmelted or partially melted particles with somewhat larger grain size. In contrast deposit C shows microspheres which are a few micrometers in diameter which result from particle splashing on impact. This is largely absent in deposit A. Single splat deposition on polished substrates analysis of all three conditions shown in Fig. 7 confirms that condition A results in a deposit with limited splashing. This is attributed to its lower overall particle temperatures which can lead to reduced splashing^53^. These observations indicate that even though deposit A does not contain a larger fraction of decomposed Ti_4_O_7_ Magneli phase there are sufficient charge carriers and reduces impedances to provide reasonable electrical conductivity. The nanoscale separations and interfaces that are still present likely contribute to the reduced thermal conductivity.
- Higher resolution XRD data collected on deposits A and C (Fig. 8) not only confirms the phase results from Fig. 1, but also suggests lower peak broadening in deposit A associated with greater fraction of unmelted particles and fine debris in the deposit. The diffraction angle for (101) peak shown in Fig. 8 shifted from 36.05° to 35.82° as the H_2_ flow changed from 0 to 9 L min^−1^. This shift in the 2θ angle can potentially be attributed to differences in residual stresses between the coating observed earlier in the curvature based stress measurement.
- Lastly, curvature based evolving stress and modulus data reported are consistent with the microstructural observations indicating that deposit A shows excellent integrity and bonding leading to high electrical conductivity.

In summary, the results and integrated analysis presented in this paper suggest potential for considering plasma spray as both a synthesis and manufacturing tool for thermoelectric oxides. Ongoing activities are focused on building thermoelectric waste heat harvesting devices that integrate *p*-*n* junctions, sequencing the layers and junctions to provide series and parallel connections to add voltage while retaining current carrying capability enabling useful energy harvesting systems. As more promising materials are identified, the fabrication concepts can be concurrently contemplated for device engineering.

## Conclusions

The thermoelectric properties of cascaded plasma spray synthesized TiO_2−x_ having oxygen defects and a combination of rutile and Magneli phases were investigated. It was observed that use of H_2_ in the plasma forming gas allowed tunability in TiO_2−x_ stoichiometry and phase content. Deposits produced under high H_2_ conditions tended to comprise of rutile phase and reduced Magneli phases with significant oxygen deficiency, while deposits produced without any H_2_ in the plasma flame resulted in predominantly rutile phase with stoichiometry closer to TiO_2_. The introduction of oxygen deficiencies with resultant formation of Ti_4_O_7_ increases electrical conductivity *σ*, but diminishes *S*. The deposits have significantly reduced through-thickness thermal conductivities due to the presence of large number of pores and interfaces within the defected microstructure of the plasma sprayed deposits. The combined attributes of optimized phase, stoichiometry and microstructure resulted in a TiO_2−x_ material with reasonable *ZT* value reaching 0.132 at 750 K for this class of transition metal oxides.

A key benefit of the plasma spray approach is the ability to directly apply these functional oxide deposits onto thermostructural components in either blanket or patterned (either through masking or via direct writing) with the ability to integrate insulator and conductors to produce complete devices. An added embodiment is the ability to accomplish this at relatively low processing temperatures (<473 K) allowing for multilayer assembly to enable direct fabrication of thermoelectric systems on both planar and conformal waste heat systems. The results point to a pathway for further exploration of transition metal oxide based thermoelectric materials through a flexible and scalable manufacturing process.

## Methods

### Deposit preparation and particle diagnostics

Sub-stoichiometric TiO_2−x_ deposits were fabricated by atmospheric plasma spray process with torch hardware (SinplexPro^TM^, Oerlikon Metco), varying the hydrogen gas ratio. As noted in the paper, although a much larger range of plasma spray operating conditions including use of both conventional and cascaded plasma sprays were contemplated significant results were only observed for the cascaded plasma attributed to its more uniform plasma flow field. The details of spray conditions are provided in Table 1. An air jet attached to the torch and air knife below the substrate both blowing towards the substrate were employed to cool the deposit during spraying. A nominally stoichiometric spray grade feedstock powder was chosen; TiO_1.9_ (Metco 102, −45 + 11 μm, fused and crushed, Oerlikon Metco). These materials are used for wear applications and as such stoichiometry is not carefully monitored. The deposits for Seebeck and electrical conductivity measurements were deposited on YSZ (9024, −75 + 10 μm, Saint-Gobain) coated stainless steel substrate (as a dielectric) through bar-shaped masks (2.5 mm W × 140 mm L and 4 mm W × 25.4 mm L, respectively). Thermal conductivity deposits were fabricated simultaneously on 12.7 mm dia. graphite rods, which allowed for deposit-substrate delamination post-deposition. Average thickness of deposits was 250 μm for Seebeck and electrical conductivity measurements, and 0.932, 1.400, and 0.854 mm for thermal conductivity measurement, respectively. All the deposits were prepared on preheated substrates with raster speed and stand-off distance of the plasma torch set to 1000 mm s^−1^ and 100 mm, respectively. In-flight particles’ temperature and velocity were monitored by using particle diagnostic sensor (AccuraSpray-G3, Tecnar Automation Lte.)(See Table 1). Deposits were also deposited on the *In-situ* coating property (ICP) sensor, which allows simultaneous measurement of substrate curvature and substrate temperature^42^.

### Microstructure and Phase Analysis

Cross-section microstructure of as-sprayed deposits and surface microstructure of splats and fractured deposits were investigated by field-emission scanning electron microscopy (FE-SEM, LEO 1550, Zeiss). X-ray diffraction of the feedstock, and deposits was carried out on X-ray diffractometer (XRD, D8 Discover, Bruker) in vertical Bragg-Brentano geometry (2.5° Soller slits in both primary and secondary beam and 0.5° divergence slit in primary path) with filtered CuK*α* radiation (Ni *β* filter in secondary path). Since linear 1D detector was used, beam knife was placed above the samples in order to minimize the detection of air scattering. Additional measurement using High resolution XRD (Ultima III, Rigaku) was performed, with a Cu-Kα radiation source at 40 kV and 44 mA between 2 θ values of 10° and 80°. Thermogravimetric analysis was performed in order to measure stoichiometry of powder, and the deposits using a thermal balance (TG/DSC, STA 449C, Netzsch) while heating TiO_2−x_ deposits up to 1073 K at 10 K min^−1^, holding it for 30 minutes in air to fully oxidize. The weight of the TiO_2−x_ deposits was approximately 30 mg.

### Thermoelectric Characterization

Voltage and electrical resistivity of the TiO_2−x_ deposits were measured using digital multimeter (Keithley 2700). Seebeck Coefficient was calculated using the measured voltage generated from temperature gradient through each end of TiO_2−x_ deposits, heating one end of the substrate with propane gas torch while cooling the other end with cold air gun (Vortex). Pt/chromel wire was measured as a reference for Seebeck coefficient measurement shown in Fig. 3(b). The deposits were junction with Pt wires using Ag paste for the measurements. Data obtained with our technique showed good match to benchmark literature information on these materials. K-type thermocouples were directly attached on each end of deposit surface to measure temperature. Electrical conductivity was measured by four-point probe technique from room temperature to 750 K. More about this measurement can be found in Han S. *et al*.^54^. Thermal conductivity was measured using laser flashing method (FLASHLINE^TM^ SYSTEM X-PLATFORM^TM^, Anter Corporation) from room temperature to 750 K. Of those measurements, density was measured using the Archimedes method. Electrical conductivity and Seebeck coefficient were measured in-plane direction and thermal conductivity was measured in through-thickness direction. This is because it is very difficult to accurately measure in-plane thermal conductivity of sprayed materials as they have a very small dimension in the thickness axis. Our past work^49^ has shown that there is about 10–20% discrepancy in the two orientations, but they tend to be self-consistent, that is if the conductivity in one orientation is higher so is the other orientation. This is because plasma sprayed structures are built by thousands of splats and chaotic assembly to some extent normalizes the anisotropy correlation to microstructure.

## Additional Information

**How to cite this article**: Lee, H. *et al*. Thermoelectric properties of *in-situ* plasma spray synthesized sub-stoichiometry TiO_2−x_. *Sci. Rep.* **6**, 36581; doi: 10.1038/srep36581 (2016).

**Publisher’s note:** Springer Nature remains neutral with regard to jurisdictional claims in published maps and institutional affiliations.

## Figures and Tables

**Table 1:** Cascaded plasma sprayed parameters for deposition of the TiO_2−x_.

Deposit	Stoichiometry	Feed rate (g min^−1^)	Ar flow (L min^−1^)	H_2_ flow (L min^−1^)	Current (A)	Power (KW)	Mean particle velocity (m s^−1^)	Mean particle temperature (^°^C)	Density (g cm^−3^)
A	TiO_1.93_	30	45	0	380	29.3	230 ± 2	2540 ± 24	3.925
B	TiO_1.90_	30	45	3	380	32.8	240 ± 2	2766 ± 21	4.138
B*	TiO_1.86_	30	45	6	380	35.5	241 ± 4	3040 ± 66	4.185
C	TiO_1.83_	30	45	9	380	37.2	239 ± 3	3539 ± 123	4.218

Deposit B* is included to show the trend in stoichiometry affected by the process condition.

## References

[b1] Wood C. Materials for thermoelectric energy conversion. Rep. Prog. Phys. 51, 459 (1988).

[b2] Snyder G. J. & Toberer E. S. Complex thermoelectric materials. Nat. Mater 7, 105–114 (2008). 18219332 10.1038/nmat2090

[b3] DiSalvo F. J. Thermoelectric cooling and power generation. Science 285, 703–706 (1999). 10426986 10.1126/science.285.5428.703

[b4] Heremans J. P. . Enhancement of thermoelectric efficiency in PbTe by distortion of the electronic density of states. Science 321, 554–557 (2008). 18653890 10.1126/science.1159725

[b5] Wang H., Pei Y., LaLonde A. D. & Snyder G. J. Heavily Doped p‐Type PbSe with High Thermoelectric Performance: An Alternative for PbTe. Adv. Mater. 23, 1366–1370 (2011). 21400597 10.1002/adma.201004200

[b6] Wang X. . Enhanced thermoelectric figure of merit in nanostructured n-type silicon germanium bulk alloy. Appl. Phys. Lett. 93, 193121 (2008). 10.1021/nl8026795 19367858

[b7] Zaitsev V. . Highly effective Mg 2 Si 1−x Sn x thermoelectrics. Phys. Rev. B 74, 045207 (2006).

[b8] Sales B., Mandrus D. & Williams R. K. Filled skutterudite antimonides: a new class of thermoelectric materials. Science 272, 1325 (1996). 8662465 10.1126/science.272.5266.1325

[b9] Shi X. . On the Design of High‐Efficiency Thermoelectric Clathrates through a Systematic Cross‐Substitution of Framework Elements. Adv. Funct. Mater. 20, 755–763 (2010).

[b10] Gascoin F., Ottensmann S., Stark D., Haïle S. M. & Snyder G. J. Zintl phases as thermoelectric materials: tuned transport properties of the compounds CaxYb1–xZn2Sb2. Adv. Funct. Mater. 15, 1860–1864 (2005).

[b11] Yang J. . Evaluation of Half‐Heusler Compounds as Thermoelectric Materials Based on the Calculated Electrical Transport Properties. Adv. Funct. Mater. 18, 2880–2888 (2008).

[b12] Pickett W. E. Electronic structure of the high-temperature oxide superconductors. Rev. Mod. Phys. 61, 433 (1989).

[b13] Koumoto K., Wang Y., Zhang R., Kosuga A. & Funahashi R. Oxide thermoelectric materials: a nanostructuring approach. Annu. Rev. Mater. Res. 40, 363–394 (2010).

[b14] Ohta S., Ohta H. & Koumoto K. Grain size dependence of thermoelectric performance of Nb-doped SrTiO3 polycrystals. J. Ceram. Soc. Jpn. 114, 102–105 (2006).

[b15] Walia S. . Transition metal oxides–Thermoelectric properties. Prog. Mater. Sci. 58, 1443–1489 (2013).

[b16] Matsui M. & Akaogi M. Molecular dynamics simulation of the structural and physical properties of the four polymorphs of TiO2. Mol. Simul. 6, 239–244 (1991).

[b17] He Q. . Thermoelectric property studies on bulk TiOx with x from 1 to 2. Appl. Phys. Lett. 91, 052505 (2007).

[b18] Andersson S., Collen B., Kuylenstierna U. & Magnéli A. Phase analysis studies on the titanium-oxygen system. Acta Chem. Scand. 11, 1641–1652 (1957).

[b19] Itakura M., Niizeki N., Toyoda H. & Iwasaki H. Hall effect and thermoelectric power in semiconductive TiO2. Jpn. J. Appl. Phys. 6, 311 (1967).

[b20] Lu Y., Hirohashi M. & Sato K. Thermoelectric properties of non-stoichiometric titanium dioxide TiO2−x fabricated by reduction treatment using carbon powder. Mater. Trans. 47, 1449–1452 (2006).

[b21] Tsuyumoto I., Hosono T. & Murata M. Thermoelectric power in nonstoichiometric orthorhombic titanium oxides. J. Am. Ceram. Soc. 89, 2301–2303 (2006).

[b22] Tang J., Wang W., Zhao G.-L. & Li Q. Colossal positive Seebeck coefficient and low thermal conductivity in reduced TiO2. J. Phys. Condens. Matter 21, 205703 (2009). 21825536 10.1088/0953-8984/21/20/205703

[b23] Sharma A., Gouldstone A., Sampath S. & Gambino R. J. Anisotropic electrical conduction from heterogeneous oxidation states in plasma sprayed TiO2 coatings. J. Appl. Phys. 100, 114906 (2006).

[b24] Colmenares-Angulo J., Cannillo V., Lusvarghi L., Sola A. & Sampath S. Role of process type and process conditions on phase content and physical properties of thermal sprayed TiO2 coatings. J. Mater. Sci. 44, 2276–2287 (2009).

[b25] Zhang L. W., Fu H. B. & Zhu Y. F. Efficient TiO2 photocatalysts from surface hybridization of TiO2 particles with graphite‐like carbon. Adv. Funct. Mater. 18, 2180–2189 (2008).

[b26] Chang L., Zhang Z., Breidt C. & Friedrich K. Tribological properties of epoxy nanocomposites: I. Enhancement of the wear resistance by nano-TiO2 particles. Wear 258, 141–148 (2005).

[b27] Herman H., Sampath S. & McCune R. Thermal spray: current status and future trends. MRS Bulletin 25, 17–25 (2000).

[b28] Sampath S. Thermal spray applications in electronics and sensors: past, present, and future. J. Therm. Spray Technol. 19, 921–949 (2010).

[b29] Solonenko O. P. & Smirnov A. V. Advanced oxide powders processing based on cascade plasma. JPCS. 550, 012017 (2014).

[b30] Ueno K. . In Thermoelectrics, 1998. Proceedings ICT 98. XVII International Conference on. 418–421 (IEEE).

[b31] Fu G., Zuo L., Longtin J., Nie C. & Gambino R. Thermoelectric properties of magnesium silicide fabricated using vacuum plasma thermal spray. J. Appl. Phys. 114, 144905 (2013).

[b32] Fu G. . Thermoelectric properties of magnesium silicide deposited by use of an atmospheric plasma thermal spray. J. Electron. Mater. 43, 2723–2730 (2014).

[b33] Goldsmid H. & Douglas R. The use of semiconductors in thermoelectric refrigeration. Br. J. Appl. Phys. 5, 386 (1954).

[b34] Fergus J. W. Oxide materials for high temperature thermoelectric energy conversion. J. Eur. Cera. Soc. 32, 525–540 (2012).

[b35] Telkes M. Solar thermoelectric generators. J. Appl. Phys. 25, 765–777 (1954).

[b36] Riffat S. B. & Ma X. Thermoelectrics: a review of present and potential applications. Appl. Therm. Eng. 23, 913–935 (2003).

[b37] Sampath S. & Wayne S. Microstructure and properties of plasma-sprayed Mo-Mo2C composites. J. Therm. Spray Technol. 3, 282–288 (1994).

[b38] Vardelle A., Fauchais P. & Themelis N. Oxidation of metal droplets in plasma sprays. (ASM International, Materials Park, OH (United States), 1995).

[b39] Kuroda S., Fukushima T. & Kitahara S. Significance of quenching stress in the cohesion and adhesion of thermally sprayed coatings. J. Therm. Spray Technol. 1, 325–332 (1992).

[b40] Kulkarni A. . Comprehensive microstructural characterization and predictive property modeling of plasma-sprayed zirconia coatings. Acta Mater. 51, 2457–2475 (2003).

[b41] Kuroda S., Fukushima T. & Kitahara S. Simultaneous measurement of coating thickness and deposition stress during thermal spraying. Thin solid films 164, 157–163 (1988).

[b42] Matejicek J. & Sampath S. *In situ* measurement of residual stresses and elastic moduli in thermal sprayed coatings: Part 1: apparatus and analysis. Acta Mater. 51, 863–872 (2003).

[b43] Sampath S., Srinivasan V., Valarezo A., Vaidya A. & Streibl T. Sensing, control, and *in situ* measurement of coating properties: an integrated approach toward establishing process-property correlations. J. Therm. Spray Technol. 18, 243–255 (2009).

[b44] Valarezo A., Choi W. B., Chi W., Gouldstone A. & Sampath S. Process Control and Characterization of NiCr Coatings by HVOF-DJ2700 System: A Process Map Approach. J. Therm. Spray Technol. 19, 852–865 (2010).

[b45] Mikami M. & Ozaki K. Thermoelectric properties of nitrogen-doped TiO2−x compounds. JPCS. 379, 012006 (2012)

[b46] Houlihan J. F., Danley W. & Mulay L. Magnetic susceptibility and EPR spectra of titanium oxides: Correlation of magnetic parameters with transport properties and composition. J. Solid State Chem. 12, 265–269 (1975).

[b47] Conze S. . Magnéli phases Ti4O7 and Ti8O15 and their carbon nanocomposites via the thermal decomposition-precursor route. J. Solid State Chem. 229, 235–242 (2015).

[b48] Harada S., Tanaka K. & Inui H. Thermoelectric properties and crystallographic shear structures in titanium oxides of the Magneli phases. J. Appl. Phys. 108, 083703 (2010).

[b49] Fuda K., Shoji T., Kikuchi S., Kunihiro Y. & Sugiyama S. Fabrication of Titanium Oxide-Based Composites by Reactive SPS Sintering and Their Thermoelectric Properties. J. Electron. Mater. 42, 2209–2213 (2013).

[b50] Liu C. . Chemical Tuning of TiO2 Nanoparticles and Sintered Compacts for Enhanced Thermoelectric Properties. J. Phys. Chem. C 117, 11487–11497 (2013).

[b51] Kumar G., Prasad G. & Pohl R. Experimental determinations of the Lorenz number. J. Mater. Sci. 28, 4261–4272 (1993).

[b52] Chi W., Sampath S. & Wang H. Ambient and high-temperature thermal conductivity of thermal sprayed coatings. J. Therm. Spray Technol 15, 773–778 (2006).

[b53] Sampath S., Jiang X., Matejicek J., Leger A. & Vardelle A. Substrate temperature effects on splat formation, microstructure development and properties of plasma sprayed coatings Part I: Case study for partially stabilized zirconia. Mat. Sci. Eng. A-Struct. 272, 181–188 (1999).

[b54] Han S. J., Chen Y. & Sampath S. Role of process conditions on the microstructure, stoichiometry and functional performance of atmospheric plasma sprayed La(Sr)MnO3 coatings. J. Power Sourecs 259, 245–254 (2014).

